# Clinical characteristics, complications and outcomes of critically ill patients with Dengue in Brazil, 2012-2024: A nationwide, multicenter cohort study

**DOI:** 10.1016/j.ijid.2025.108023

**Published:** 2025-08-15

**Authors:** Igor Tona Peres, Otavio T. Ranzani, Leonardo S.L. Bastos, Silvio Hamacher, Tom Edinburgh, Esteban Garcia-Gallo, Fernando Augusto Bozza

**Affiliations:** 1 Department of Industrial Engineering (DEI), Pontifical Catholic University of Rio de Janeiro, Rio de Janeiro, Brazil; 2 ISARIC, Oswaldo Cruz Foundation (FIOCRUZ), Rio de Janeiro, Brazil; 3 Institut de Recerca Sant Pau (IR SANT PAU), Barcelona, Spain; 4 Pulmonary Division, Faculty of Medicine, Heart Institute, Hospital das Clínicas da Faculdade de Medicina da Universidade de São Paulo, São Paulo, Brazil; 5 ISARIC, Pandemic Sciences Institute, University of Oxford, Oxford, UK; 6 National Institute of Infectious Disease Evandro Chagas (INI), FIOCRUZ, RJ, Brazil; 7 D’Or Institute for Research and Education, Rio de Janeiro, Brazil; 8 CHRC, NOVA Medical School, Universidade Nova de Lisboa, Lisboa, Portugal

**Keywords:** Dengue, Intensive care, Risk factors, Clinical management, Prediction

## Abstract

**Background:**

Dengue outbreaks are a major public health issue, with Brazil reporting 71% of global cases in 2024.

**Purpose:**

This study aims to describe the profile of severe dengue patients admitted to Brazilian intensive care units (ICUs) (2012-2024), assess trends over time, describe new onset complications while in ICU, and determine the risk factors at admission to develop complications during ICU stay.

**Methods:**

We performed a prospective study of dengue patients from 253 ICUs across 56 hospitals. We used descriptive statistics to describe the dengue ICU population, logistic regression to identify risk factors for complications during the ICU stay, and a machine learning framework to predict the risk of evolving to complications. Visualizations were generated using ISARIC VERTEX.

**Results:**

Of 11,047 admissions, 1117 admissions (10.1%) evolved to complications, including non-invasive (437 admissions) and invasive ventilation (166), vasopressor (364), blood transfusion (353), and renal replacement therapy (103). Age ≥80 (odds ratio [OR]: 3.10, 95% confidence interval: 2.02-4.92), chronic kidney disease (OR: 2.94, 2.22-3.89), liver cirrhosis (OR: 3.65, 1.82-7.04), low platelets (<50,000 cells/mm^3^; OR: 2.25, 1.89-2.68), and high leukocytes (>7000 cells/mm^3^; OR: 2.47, 2.02-3.03) were significant risk factors for complications. A machine learning tool for predicting complications was proposed, showing accurate discrimination and calibration.

**Conclusion:**

We described a large cohort of dengue patients admitted to ICUs and identified key risk factors for severe dengue complications, such as advanced age, presence of comorbidities, higher level of leukocytes, and lower level of platelets. The proposed prediction tool can be used for early identification and targeted interventions to improve outcomes in dengue-endemic regions.

## Introduction

Dengue is a mosquito-borne viral infection caused by the dengue virus, transmitted primarily by *Aedes aegypti*, having four different serotypes and multiple genotypes [1]. Dengue outbreaks are frequent public health events affecting populations and health systems from numerous countries, particularly in tropical and sub-tropical regions [2]. Currently, the disease is endemic in more than 100 countries. The World Health Organization (WHO) reported over 14.3 million cases and 10,839 deaths in 2024, a sharp increase compared to the 4.6 million cases and 2400 deaths recorded in 2023 [3]. Brazil appears as the most affected country, accounting for 71% of global reported cases and 57% of deaths, with 10.2 million cases and 6239 deaths in 2024, marking the highest dengue outbreak globally [3].

Regarding severe dengue cases, the WHO reported 52,311 severe cases worldwide in 2024, with Brazil accounting for 8268 of these cases [3]. Despite the expanding outbreak, comprehensive studies on the clinical characteristics of severe dengue patients remain scarce. Existing literature has primarily focused on sociodemographic factors and general symptoms rather than detailed clinical profiles [4]. Few studies analyzed clinical risk factors of severe dengue; however, they used small cohort sizes (<200 patients) to develop the analysis [5,6]. To address this gap, this study aims to analyze the characteristics of severe dengue patients admitted to intensive care units (ICUs) over a 13-year period (2012-2024) within one of Brazil’s largest hospital networks. Additionally, it seeks to identify clinical risk factors associated with complications during ICU stays. Severe dengue can lead to critical conditions such as severe plasma leakage, hemorrhage, or organ failure, which may escalate rapidly during the critical phase of illness, requiring urgent intervention to prevent mortality [1].

The key research questions addressed in this study are: (1) What are the demographic and clinical profiles of ICU dengue patients; (2) How has this profile changed from 2012 to 2024; (3) How many patients develop complications during ICU stays; and (4) What are the risk factors at ICU admission for developing complications during ICU stay.

## Materials and methods

### Study design

This is a prospectively study considering all ICU admissions from January 2012 to June 2024. The study was conducted across 253 general medical-surgical ICUs within 56 hospitals from 14 Brazilian states, all part of an integrated hospital network (Rede D’Or-Sao Luiz), and according to the STROBE statement [7]. The study received approval from both the local Ethics Committee and the Brazilian National Ethics Committee (CAAE: 17079119.7.0000.5249), and the need for informed consent was waived due to its retrospective observational design.

### Participants and data collection

We extracted all anonymized data from an electronic system designed for ICU quality improvement and benchmarking (Epimed Monitor, Rio de Janeiro, Brazil) [8]. This database includes prospectively collected structured data on all adult ICU admissions (≥16 years old), providing comprehensive clinical information. It captures features collected at admission (first 24 h), such as patient demographics, ICU admission diagnoses, comorbidities, illness severity scores, and use of organ support; and the highest or lowest haematological values collected at first 1 h of ICU admission. We also have complications and outcomes collected during the complete ICU stay, such as use of devices, ICU and hospital mortality, as well as length of stay (LOS). Table S1 shows the data dictionary, including a complete description of each variable comprised in the study. The inclusion criteria were patients with confirmed dengue infection at ICU admission (primary or secondary diagnosis based on laboratory confirmation). In this prospectively collected database, patients are prospectively categorized by a standardized form in “main reason of ICU admission,” which reflects the primary admission diagnosis, and for other secondary reasons of ICU admission. We excluded admissions with undefined outcome (42 admissions), readmissions within 30 days (32 admissions), and patients with undefined sex (three admissions) (Figure S1). Since patient sex will be one of the model’s confounders, the exclusion of undefined sex is related to the few cases in this category.

### Outcomes

The primary outcome of interest was the presence of any complication during the ICU stay, defined as a clinically significant event requiring ICU-level intervention and prompting a clear change in management or resource utilization, such as: (1) the need for organ support (e.g., invasive mechanical ventilation, non-invasive ventilation vasopressor drugs, or renal replacement therapy); (2) any blood product transfusion (e.g., red blood cells, platelets and fresh frozen plasma); or (3) death. The secondary outcomes were 60-day ICU and in-hospital mortality, and ICU and in-hospital LOS.

### Statistical analysis

We used descriptive statistics to describe the ICU population, including demographics, illness severity, comorbidities, complications, laboratory tests, and outcomes. Categorical variables were expressed as frequencies and proportions, while continuous variables were summarized as medians and interquartile ranges (IQR). We included *P*-values to assess differences between groups (complicated vs not complicated). The Wilcoxon rank sum test was used for continuous variables, and the Pearson’s Chi-squared test was employed for categorical ones. To illustrate trends over time, we included bar and line plots showing the time series of ICU admissions and outcomes across the years.

To identify risk factors for complications during the ICU stay, we evaluated the association between patient clinical characteristics and the primary outcome. The set of features included in the analysis was selected by clinical relevance. Collinearity among variables was pre-assessed using Pearson’s correlation coefficient. Then, we used a univariable logistic regression model to estimate the individual association between each variable and the outcome. We selected the features to the multivariable logistic regression based on clinical judgment and considering the *P*-value of the univariable regression. Association was calculated by the estimated odds ratios (ORs) and their corresponding 95% confidence intervals (CIs).

We also developed a model to predict the risk of evolving dengue ICU complications following a machine learning framework [9,10]. We used stratified random sampling [11] to split 80% of the dataset for training and 20% for testing. Stratified random sampling selects a sample from a population by dividing the population into subgroups (strata) based on shared characteristics and then randomly selecting samples from each stratum. This ensures that each subgroup is adequately represented in the training and testing sample [12]. We trained and compared the logistic regression with two machine learning models (Gradient Boosting Machine and Random Forests) using the set of risk factors obtained from the previous analysis. We used 10-fold cross–validation to tune the models and evaluated them based on discrimination and calibration measures. Discrimination was measured by the area under the curve (AUC), and calibration was addressed by the calibration belts [13]. Variables with more than 30% missing data were excluded from the multivariable analysis, while the remaining variables were imputed using the Multiple Imputation by Chained Equations algorithm [14].

We performed two experiments in terms of prediction modelling: (1) the first one used the same set of features from the previous multivariable analysis; (2) the second one grouped all comorbidities into one unique feature, called “number of co-morbidities,” which has three categories (0; 1-2; 3+). We developed an open-source website including the risk calculator that could be used to generate the predictions of complications for ICU dengue patients: https://l4×2nl-igor-peres.shinyapps.io/Dengue_Risk_Calculator/. We also included the model formulation in the results in case other researchers want to apply our model in different contexts.

Plots and figures were generated using ISARIC VERTEX [15], a web-based application that produces tables and visualizations to support key research questions. VERTEX is part of the IS-ARIC Clinical Epidemiology Platform, developed to standardize and streamline data collection and analysis in outbreak-related clinical research. A public implementation of VERTEX for this study is available at: https://projects.vertex.isaric.org/?param=Dengue_ICU. The developed codes for VERTEX are publicly available at GitHub (https://github.com/ISARICResearch/VERTEX).

## Results

A total of 11,047 adult patients were included in the analysis. The median age was 45 years (IQR: 32-63), and 91% were from the emergency department (Table 1). The median SAPS-3 was 40 (36-46), and with a median SOFA of 2 (0-3). The 60-day in-hospital mortality was 1.4% (*n* = 157), and the median hospital LOS was 5 (3-7). A total of 1117 admissions (10.1%) developed complications during ICU stay, such as non-invasive ventilation (*n* = 437; 4.0%), use of vasopressor (*n* = 364; 3.3%), blood transfusion (*n* = 353; 3.2%), invasive mechanical ventilation (*n* = 166; 1.5%), renal replacement therapy (*n* = 103; 0.9%) and high-flow nasal cannula (*n* = 23; 0.2%). Hypertension (*n* = 3291; 30%), diabetes (*n* = 1644; 15%) and cardiovascular disease (*n* = 913; 8.3%) were the most prevalent comorbidities. Among the 1117 patients who developed complications during ICU stay, 364 (33%) required vasopressors or had shock at admission (first 24 h), while 311 (28%) required non-invasive ventilation. Mechanical ventilation and renal replacement therapy were less common at admission, observed in 86 (7.7%) and 60 (5.4%) patients, respectively.

**Figure 1:**
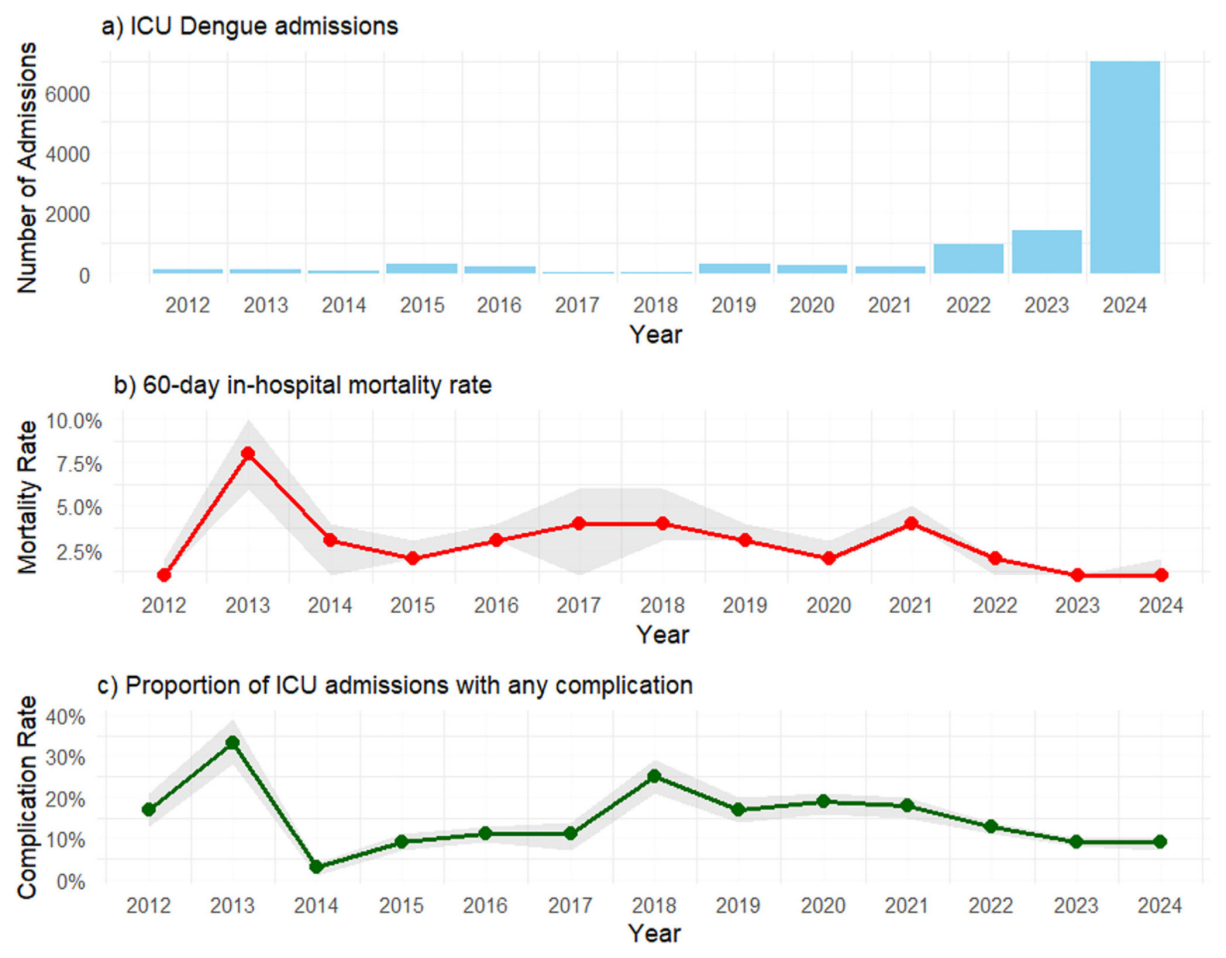
(a) Admissions and in-hospital mortality rate of ICU dengue patients over time; (b) 60-day in-hospital mortality rate; and (c) proportion of admission with any complication.

We analyzed trends in ICU admissions and mortality over time (Figure 1). The number of ICU admissions with a dengue diagnosis was higher in 2024, corresponding to almost half of the cohort size, characterizing a large dengue outbreak. In contrast, we noted a declining trend over the years when analyzing the proportion of admissions with any complication and the in-hospital mortality rate. The in-hospital mortality rate was significantly higher (*P <* 0.001) during 2012-2021 (3%) compared to 2022-2023 (0.9%) and 2024 (1.2%). Similarly, the proportion of patients with complications decreased, dropping from 16% in 2012-2021 to 10% in 2022-2023 and further to 8.7% in 2024 (*P <* 0.001). The complete profile of ICU patients over the years can be assessed in Table S2.

### Complications during ICU Stay

During the complete ICU stay, vasopressors or shock management were necessary for 430 patients (38%), while non-invasive ventilation was used in 437 patients (39%). Other complications included transfusions (353; 32%), mechanical ventilation (166; 15%), renal replacement therapy (103; 9.2%), and high-flow nasal cannula (23; 2.1%). Patients with complications had longer ICU and hospital LOS (*P <* 0.001), with a median of 4 (3-7) and 7 (5-11) days, respectively, compared to 3 (2-4) and 5 (3-6) days in patients without complications. Mortality was significantly higher in the former group (*P <* 0.001), with a 60-day ICU mortality rate of 8.7% and a 60-day hospital mortality rate of 11%, compared to 0.1% and 0.4% in patients without complications. The patients who died were older (75, 59-85), with a higher proportion coming from the ward, floor, or step-down unit (16%), presenting a greater number of comorbidities, and had higher severity scores (SAPS-3 = 57, 51-67; SOFA = 4, 2-7; 36% of frail patients). More than half of the patients who required mechanical ventilation (58%) or vasopressor (54%) died, with these two complications being the most impactful on mortality outcomes. The mortality was lower in patients with non-invasive ventilation (27%), transfusion (25%), renal replacement therapy (21%), and high-flow nasal cannula (3.8%).

Patients who developed complications in the ICU showed distinct laboratory values during the first hours of admission compared to those without complications (Table 1). Patients with any complications presented similar median lowest platelet counts at admission compared to those without (median 88,000, IQR: 52,000-139,000; vs 89,000, IQR: 37,000-151,000 cells/mm^3^; *P* = 0.2). However, patients with complications had higher leukocyte count (5100, 3200-9000; vs 3700, 2600-5800 cells/mm^3^; *P <* 0.001) and arterial lactate levels (1.64, 1.2-2.6; vs 1.4, 1.1-2.0 mmol/L; *P <* 0.001) at admission compared to those without complications. Blood pressure and creatinine showed smaller differences but consistently suggested a more severe profile among patients with complications. The lowest systolic, diastolic, and mean arterial pressures did not show significant variations. The median age was higher in the complication group (58 [38-74] vs 44 [32-61]; *P <* 0.001), and the proportion of female sex was similar (51% vs 52%; *P* = 0.5). All comorbidities were more frequent in the complication group, except asthma (*P* = 0.4).

**Figure 2:**
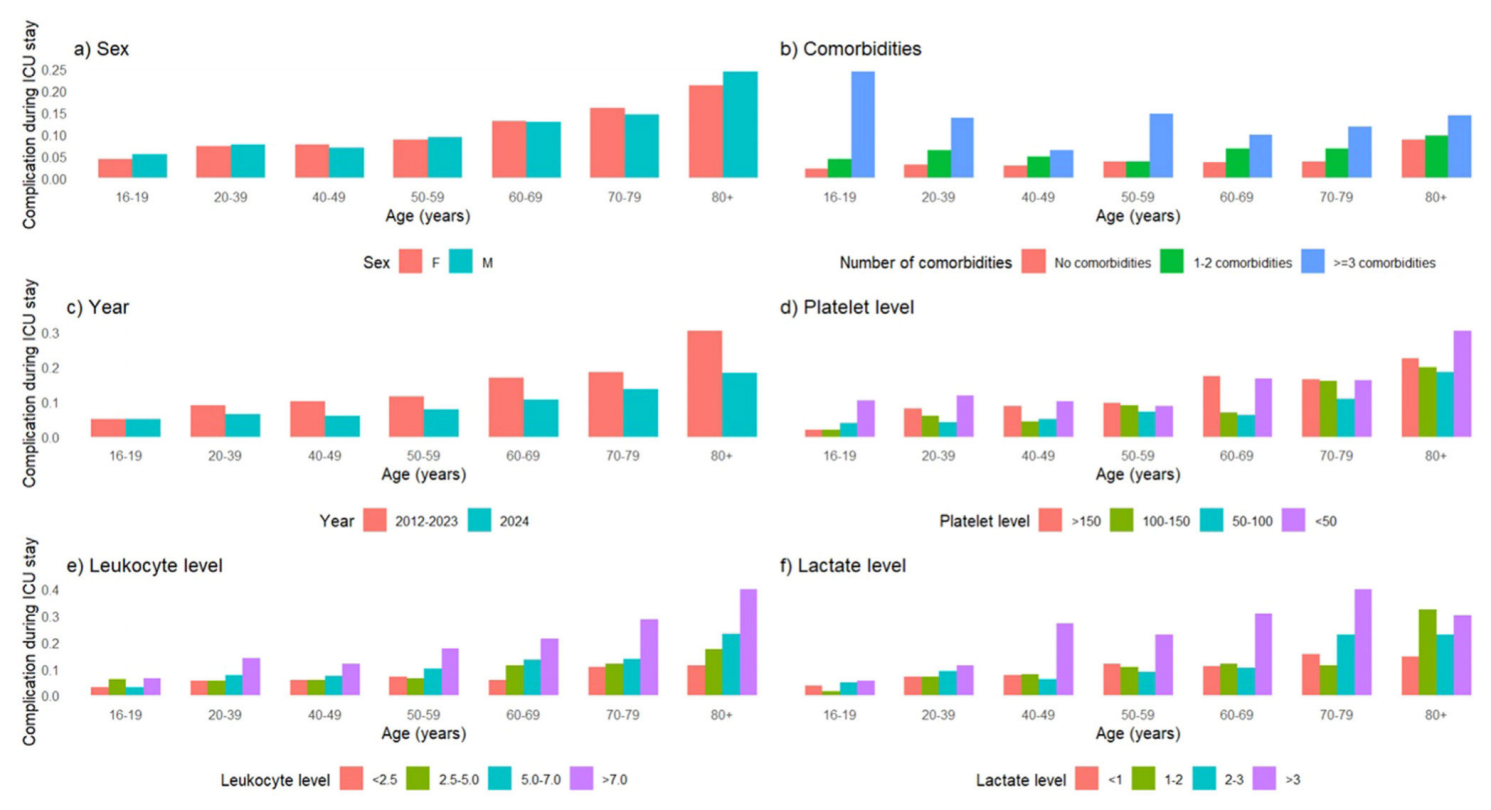
Complications by age, sex, number of comorbidities, period of time, platelet levels, leukocyte levels, and lactate levels, stratified by age groups. Relationship between complications and (a) sex, (b) comorbidities, (c) year, (d) platelets level, (e) leukocyte level, and (f) lactate level, stratified by age groups.

In Figure 2, we observe that as age and the number of comorbidities increase, so does the likelihood of developing at least one complication in the ICU (Figure 2b). The proportion of patients developing complications is greater for young adults (16-19 years old) with three or more comorbidities, and we also can note that for all ages, the higher the number of comorbidities, the greater the proportion of complications (Figure 2b). Patient sex did not appear to be related to the absolute risk of complication (Figure 2a). Additionally, admissions from 2012 to 2023 reported a greater proportion of complications than those from 2024 (Figure 2c). Figure 2d-2f also illustrates the relationship between ICU complications and platelet, leukocyte, and lactate levels (at first 1 h of admission), stratified by age groups. Across all ages, lactate levels above 3 mmol/L and leukocyte counts above 7000 cells/mm^3^ (leucocytosis) were associated with an increased risk of progression to complications (Figure 2e and 2f). Regarding platelets, although the univariate analysis of Table 1 did not reveal significant effect of platelets count using the continuous value, when we stratify in categories, we can note that levels below 50,000 cells/mm^3^ (thrombocytopenia) appeared to increase risk across most age groups (Figure 2d). The same effect could be noted when analyzing the density distribution of these three laboratory variables (Figure S2). Therefore, we could note that patients with thrombocytopenia, leuco-cytosis, and elevated lactate at baseline (first 1 h of ICU admission) were at risk of additional or new complications during ICU stay.

We also analyzed the characteristics of these admissions, which appears to be in the higher risk group. Compared to the overall population, the patients who presented platelets lower than 50,000 cells/mm^3^ had greater proportion of new complications (13.6%) and similar age composition (45, 33-61) and slightly higher severity scores (SAPS-3 = 45, 42-50; SOFA = 3, 3-4; 5.6% of frail patients). The most prevalent complication was the blood transfusion (8.8%), followed by vasopressor use or shock (3.2%), non-invasive ventilation (2.6%), mechanical ventilation (1.4%), renal replacement therapy (0.9%), and high-flow nasal cannula (<0.1%). The ICU mortality rate (1.1%) and ICU LOS (3, 2-4) for this subgroup of thrombocytopenia patients were similar to the general population (1.0% and 3[2, 4], respectively).

Regarding the patients who presented leukocyte counts above 7000 cells/mm^3^, they presented greater risk of becoming complicated (18.4%) and presented similar age composition (45, 32-65) and severity scores (SAPS-3 = 42, 37-49; SOFA = 2, 0-3; 11% of frail patients). The most prevalent complication was non-invasive ventilation (8.0%) followed by vasopressor use or shock (7.9%), blood transfusion (5.2%), mechanical ventilation (4.6%), renal replacement therapy (2.7%), and high-flow nasal cannula (0.5%). The ICU mortality rate (2.9%) for this subgroup of leucocytosis patients were higher compared to the general population (1.0%); however, the ICU LOS presented similar statistics (3, 2-5).

### Risk factors of ICU complications during ICU stay

Table 2 shows the univariable and multivariable logistic regression to investigate the relationship between demographic, clinical, and laboratory variables and the risk of developing complications in the ICU. The features included in the model did not present notable collinearity (Figure S3). We observed in the univariable model that older age (80+, OR: 5.59, 95% CI: 3.77-8.61; *P <* 0.001); the presence of chronic kidney disease (OR: 5.12, 3.96-6.58; *P <* 0.001), liver cirrhosis (OR: 4.49, 2.35-8.24; *P <* 0.001), cerebrovascular disease (OR: 3.20, 2.51-4.06; *P <* 0.001), obesity (OR: 2.32, 1.67-3.15; *P <* 0.001) and immunosuppression (OR: 2.76, 2.03-3.69; *P <* 0.001) were independently associated with complications. Additionally, low platelet counts (<50,000 cells/mm^3^) (OR: 2.12, 1.79-2.51; *P <* 0.001), and high leukocyte counts (>7000 cells/mm^3^, OR: 3.43, 2.82-4.18; *P <* 0.001) were independently associated with the primary outcome.

**Figure 3:**
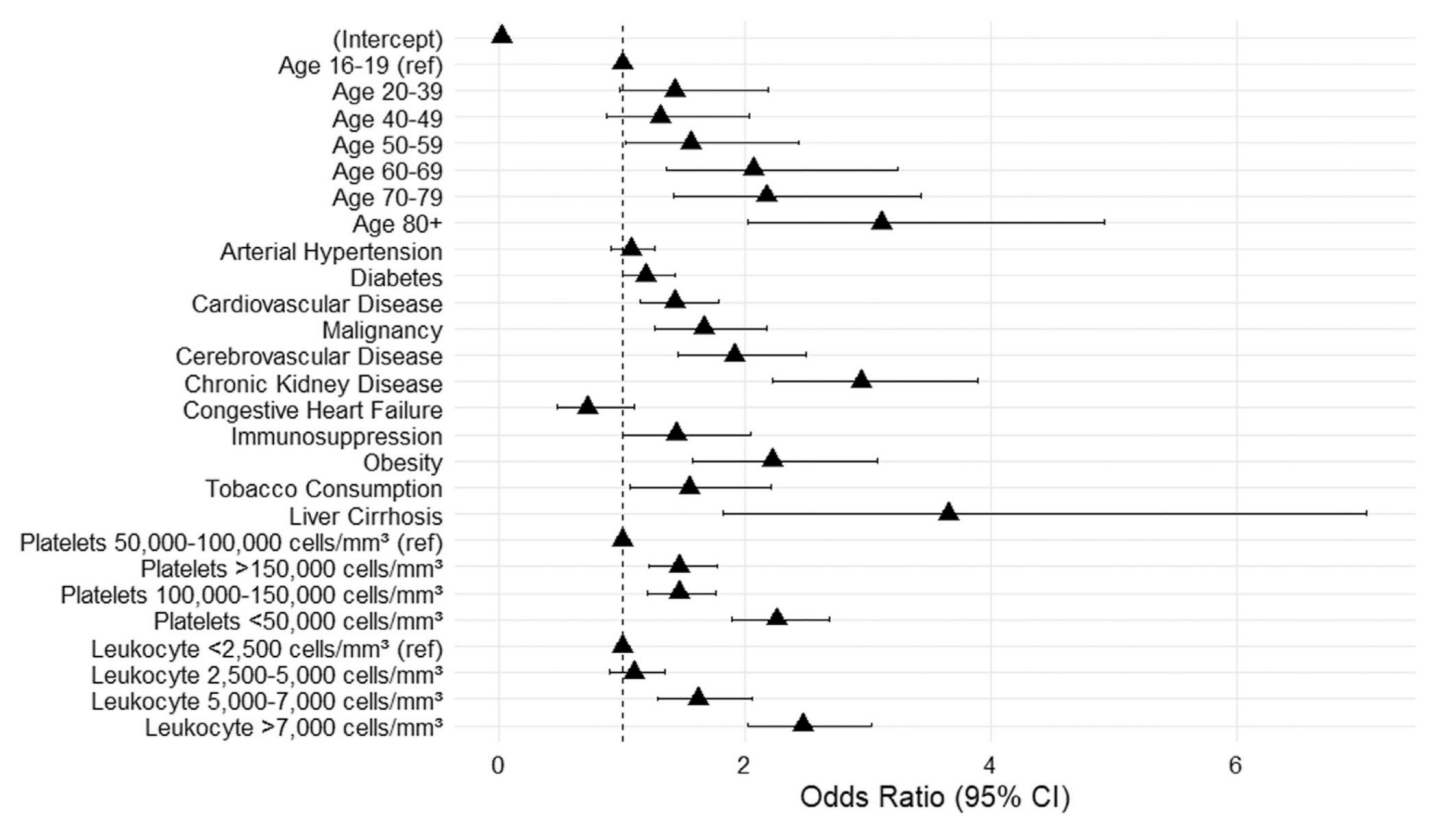
Forest plot for the multivariable logistic regression to analyze the influence of risk factors into dengue complication.

Multivariable logistic regression presents adjusted results by potential confounders. Age remained significantly related to the risk of complications, with older patients (80+) showing a progressively higher OR (3.10, 2.02-4.92; *P <* 0.001), compared to the reference group (16-19 years). The following comorbidities were significant associated with risk of complications: liver cirrhosis (OR: 3.65, 1.82-7.04; *P <* 0.001), chronic kidney disease (OR: 2.94, 2.22-3.89; *P <* 0.001), obesity (OR: 2.22, 1.55-3.08; *P <* 0.001), cere-brovascular disease (OR: 1.91, 1.46-2.49; *P <* 0.001), malignancy (OR: 1.66, 1.26-2.18; *P <* 0.001), tobacco consumption (OR: 1.55, 1.06-2.21; *P* = 0.02), cardiovascular disease (OR: 1.43, 1.14-1.78; *P* = 0.002), diabetes (OR: 1.19, 1.00-1.42; *P* = 0.047), and immuno-suppression (OR: 1.44. 1.00-2.05; *P* = 0.043). Among laboratory variables, lower platelet counts (<50,000 cells/mm^3^) and elevated leukocyte counts (>7000 cells/mm^3^) significantly (*P <* 0.001) increased the odds of complications, with ORs of 2.25 (1.89-2.68) and 2.47 (2.02-3.03), respectively. Figure 3 summarizes the multivariable logistic results. The findings highlight age, specific comorbidities, and laboratory abnormalities as key predictors of dengue complications.

### Prediction model for ICU complications during ICU stay

The prediction model using experiment (1) achieved the following results when applied to the testing dataset: Random Forests (AUC = 0.719); GBM (AUC = 0.724); logistic regression (AUC = 0.718); and considering experiment (2): Random Forests (0.711); GBM (0.713); logistic regression (0.711). The models presented accurate calibration, as can be seen in Figures S4 and S5. To simplify the application process for intensivists, since discrimination and calibration had a small difference between models and between experiments, we propose a simpler model to predict the risk to evolve to dengue complications using Equation (1) of the logistic regression model of experiment (2).

(1) $$\begin{array}{l} \log it(\hat{p})=-3.35461+Age_{class20-39}\times 0.31409+Age_{class40-49}\\ \;\times 0.19208+Age_{class50-59}\times 0.30071+Age_{class60-69}\\ \;\times 0.51040+Age_{class70-79}\times 0.55154+Age_{class>80}\\ \;\times 0.97917+Platelets_{class100-150}\times 0.04042\\ \;+Platelets_{class50-100}\times (-0.29755)+Platelets_{class<50}\\ \;\times 0.44430+Leukocyte_{class2.5-5}\times 0.05018\\ \;+Leukocyte_{class5.0-7.0}\times 0.52003+Leukocyte_{class>7.0}\\ \;\times 0.88603+N_{comorbidities1-2}\times 0.53722\\ \;+N_{comorbidities3}\times 1.23915 \end{array}$$

## Discussion

Our study provides a comprehensive analysis of the clinical and demographic profiles of dengue patients admitted to Brazilian ICUs between 2012 and 2024, identifying key risk factors for complications. With a sample size of over 11,000 patients, this represents one of the largest datasets on dengue in critically ill populations.

We identified several significant risk factors for complications in ICU dengue patients, including advanced age, associated co-morbidities (chronic kidney disease, liver cirrhosis, obesity, cerebrovascular disease, malignancy, tobacco consumption, cardiovascular disease, diabetes, and immunosuppression), and laboratory markers (low platelet levels and elevated leukocyte levels). These findings are consistent with those of Copaja-Corzo et al. [5] and Carras et al. [6]. Copaja-Corzo et al. [5] identified cardiovascular disease, elevated liver enzymes, and hypofibrinogenemia as key predictors of severe dengue. Comorbidities such as liver cirrhosis are commonly associated with elevated liver enzymes levels. Additionally, comorbidities such as liver cirrhosis, chronic kidney disease, and immunosuppression are usually related to hypofibrinogenemia. Carras et al. [6] highlighted the history of cardiovascular disease, the delayed hospital presentation (time from first symptom to hospital consultation over 2 days), and being of Western European origin as significant predictors for severe dengue.

Our findings on the predictive value of low platelet counts and high leukocyte levels align with those of Thach et al. [16] and Moallemi et al. [17], literature reviews who emphasized the importance of haematological markers, including platelet counts and liver enzymes, in dengue prognosis. However, our study goes further by integrating these biomarkers into a machine learning model to predict complications, achieving an AUC of 0.71. This approach provides a more dynamic and actionable tool for clinicians, particularly in resource-limited settings where early identification of high-risk patients is crucial.

The reviews of Chagas et al. [18] and Díaz-Quijano [19] also identified advanced age, comorbidities (such as diabetes and cardiovascular disease), and organ dysfunction (particularly liver and kidney dysfunction) as significant predictors of mortality and bleeding complications, respectively. These findings are consistent with our results, where low platelet levels and elevated liver enzymes were associated with complications. While Díaz-Quijano [19] specifically focused on bleeding complications, our study aligns with their findings on the importance of platelet counts in predicting severe outcomes. Paraná et al. [4] systematic reviewed and meta-analyzed risk factors associated with severe dengue in Latin America and indicated that secondary dengue infection, female sex, white race, and specific signs and symptoms were significant predictors of severe dengue outcomes. However, the review identified a lack of studies focusing on clinical characteristics related to severe dengue.

Interestingly, our findings on risk factors for severe dengue complications share similarities with those of Kallas et al. [20] in their study on predictors of mortality in patients with yellow fever. Both studies identified advanced age, liver dysfunction, and renal failure as significant predictors of poor outcomes. However, Kallas et al. [20] also emphasized the role of coagulopathy and elevated bilirubin levels, which were not as prominently featured in our study. This difference may reflect the distinct pathophysiological mechanisms of dengue and yellow fever, with yellow fever being more likely to cause severe hepatic injury and coagulopathy.

Our analysis of trends over time revealed a significant increase in dengue admissions in 2024, coinciding with a global surge in cases reported by the WHO [3]. Despite this increase, we observed a decline in complication and mortality rates, which may reflect improvements in ICU management and early intervention strategies. This finding contrasts with Copaja-Corzo et al. [5], who reported higher mortality rates in their cohort (8.6%), possibly due to differences in healthcare infrastructure, resource availability, and the severity of dengue strains between Brazil and Peru. Other studies have also analyzed temporal trends in dengue mortality. Shepard et al. [21] conducted a comprehensive analysis of dengue mortality trends across multiple countries, highlighting significant regional variations in mortality rates. They found that while some regions experienced declining mortality due to improved healthcare access and public health interventions, others saw stable or increasing mortality rates, particularly in areas with limited resources. Similarly, Limkittikul et al. [22] analyzed dengue mortality trends in Thailand over a 20-year period, reporting a gradual decline in mortality rates due to better disease management and public health measures. However, they noted periodic spikes in mortality during severe outbreaks.

Our machine learning model, which predicts complications including few features and with an AUC of 0.71, represents a significant advancement in dengue risk stratification. Several other studies have also explored the use of machine learning in dengue prognosis. Most of them analyzed the prediction of severe dengue for hospitalized patients or the risk of ICU admission. Caicedo-Torres et al. [23] demonstrated the utility of machine learning in identifying early markers of dengue severity for hospitalized paediatric patients, developing a model to predict the risk of ICU admission, while Huang et al. [24] highlighted the importance of combining demographic and laboratory data for accurate risk prediction of severe dengue in hospitalized patients (using WHO 2009 classification). Madewell et al. [25] had the same objective and used clinical and environmental data to predict severe dengue for hospitalized patients, while Sarma et al. [26] explored various machine learning algorithms for dengue prediction for hospitalized patients, showcasing the versatility of these approaches. Our study aligns with the broader trend of using advanced machine learning techniques to improve dengue risk stratification; however, our focus regards to ICU patients. The severe dengue literature lacks in studies analyzing critical ill patients. The identification of key risk factors, such as advanced age, chronic comorbidities, and abnormal laboratory markers, and the use of our proposed model integrated into clinical decision-support systems, which predicts complications with reasonable accuracy, can be used for early identification of high-risk patients in ICUs. This is particularly relevant in dengue-endemic regions, where timely intervention can significantly reduce mortality and ICU resource utilization.

One of the strengths of our study is the large, nationally representative dataset, which provides granular insights into the critical care context of dengue management. Our machine learning model shows promise, although further validation in external cohorts is needed to ensure its generalizability. We have the following limitations: (1) we analyzed a cohort of private ICU patients, which may have different presentation and risk factors than more general populations of dengue patients. However, we showed data from a large network of hospitals with adequate resource availability; and (2) our definition focuses on objective, clinically significant events—namely, the need for organ support, blood transfusions, and death—which capture complications relevant to ICU-level care. These outcomes partially overlap with the WHO definition of severe dengue [1], which is widely used for triage and early severity stratification in dengue. However, its direct application in the ICU setting is limited, as it was not specifically designed to reflect the clinical course and outcomes of critically ill patients. We believe that clinical interventions (e.g., transfusion) provide a more actionable and meaningful marker of disease severity than laboratory thresholds alone. We acknowledge that this approach may reduce external generalizability. At the same time, we believe it provides a useful framework for characterizing clinical severity in ICU patients and could serve as a foundation for future studies aiming to validate ICU-specific severity definitions in dengue.

Future research should focus on validating our predictive model in diverse settings and exploring the impact of targeted interventions on patient outcomes, such as the ICU datasets of ISARIC hubs in Colombia and Pakistan. Additionally, studies should investigate the role of emerging biomarkers and genetic factors in dengue prognosis, which could further refine risk stratification and treatment strategies. Moreover, future studies should also evaluate the impact of new dengue vaccines approved to be used (e.g., QDenga in Brazil) in the incidence, death and in-hospital mortality.

## Conclusion

We described a large cohort of dengue patients admitted to ICUs and identified key risk factors for severe dengue complications, such as advanced age, presence of comorbidities, elevated level of leukocytes, and low platelets levels. The proposed prediction tool can be used for early identification and targeted interventions to improve outcomes in dengue-endemic regions. By integrating clinical and laboratory data into predictive models, we provide a foundation for more effective and personalized care strategies, which are urgently needed in the face of increasing dengue burden worldwide. As dengue continues to pose a major public health challenge, particularly in endemic regions, the insights from this study can inform both clinical practice and public health strategies to mitigate its impact.

## Supplementary Material

Supplementary Information

## Figures and Tables

**Table 1:** Profile of ICU dengue admissions.

Feature	Overall *N =* 11,047	Any complication during ICU stay		*P*-value
		No *N =* 9930 (89.9%)	Yes *N =* 1117 (10.1%)	
Demographics				
Age, years	45 (32, 63)	44 (32, 61)	58 (38, 74)	<0.001
Sex (female)	5743 (52%)	5173 (52%)	570 (51%)	0.5
**Comorbidities**				
Hypertension	3291 (30%)	2805 (28%)	486 (44%)	<0.001
Diabetes	1644 (15%)	1375 (14%)	269 (24%)	<0.001
Cardiovascular disease	913 (8.3%)	740 (7.5%)	173 (15%)	<0.001
Malignancy	458 (4.1%)	357 (3.6%)	101 (9.0%)	<0.001
Cerebrovascular disease	375 (3.4%)	280 (2.8%)	95 (8.5%)	<0.001
Asthma	306 (2.8%)	271 (2.7%)	35 (3.1%)	0.4
Chronic kidney disease	281 (2.5%)	183 (1.8%)	98 (8.8%)	<0.001
Congestive heart failure	198 (1.8%)	158 (1.6%)	40 (3.6%)	<0.001
Immunosuppression	256 (2.3%)	197 (2.0%)	59 (5.3%)	<0.001
Obesity	247 (2.2%)	197 (2.0%)	50 (4.5%)	<0.001
Active smoker	227 (2.1%)	186 (1.9%)	41 (3.7%)	<0.001
Liver cirrhosis	45 (0.4%)	30 (0.3%)	15 (1.3%)	<0.001
**Admission**				
Admission source name				<0.001
Emergency room	10,014 (91%)	9108 (92%)	906 (81%)	
Ward/floor/step down unit	674 (6.1%)	534 (5.4%)	140 (13%)	
Other	355 (3.2%)	285 (2.9%)	70 (6.3%)	
Modified frailty index (MFI)				<0.001
Non-frail (MFI = 0)	6274 (57%)	5818 (59%)	456 (41%)	
Pre-frail (MFI = 1-2)	3888 (35%)	3444 (35%)	444 (40%)	
Frail (MFI ≥ 3)	885 (8.0%)	668 (6.7%)	217 (19%)	
Saps-3, points	40 (36, 46)	40 (35, 45)	47 (41, 56)	<0.001
Sofa, points	2 (0, 3)	2 (0, 3)	3 (1, 5)	<0.001
Period				<0.001
2012-2021	1682 (15%)	1418 (14%)	264 (24%)	
2022-2023	2372 (21%)	2124 (21%)	248 (22%)	
2024	6993 (63%)	6388 (64%)	605 (54%)	
**Laboratory evaluation at first admission hour**				
Lowest platelets count (cells/mm^3^)	88,000 (51,000, 140,000)	88,000 (52,000, 139,000)	89,000 (37,000, 151,000)	0.2
Highest leukocyte count (cells/mm^3^)	3800 (2600, 6100)	3700 (2600, 5800)	5100 (3200, 9000)	<0.001
Highest arterial lactate (mmol/L)	1.40 (1.10, 2.00)	1.40 (1.10, 2.00)	1.64 (1.20, 2.60)	<0.001
Bun	11 (8, 15)	11 (7, 15)	15 (10, 24)	<0.001
Highest creatinine	0.80 (0.64, 1.01)	0.80 (0.64, 1.00)	0.90 (0.70, 1.25)	<0.001
Lowest systolic blood pressure	120 (108, 132)	120 (108, 131)	120 (107, 135)	0.07
Lowest diastolic blood pressure	71 (63, 80)	71 (63, 80)	71 (61, 80)	0.12
Lowest mean arterial pressure	88 (79, 96)	87 (79, 96)	88 (77, 98)	0.7
Lowest PaO2FiO2	143 (67, 310)	138 (62, 310)	157 (100, 292)	0.003
Lowest Glasgow coma scale	15 (15, 15)	15 (15, 15)	15 (15, 15)	<0.001
Highest heart rate	75 (66, 85)	75 (66, 85)	78 (68, 89)	<0.001
Highest respiratory rate	18 (16, 20)	18 (16, 20)	18 (16, 20)	<0.001
Highest bilirubin	0.50 (0.31, 0.70)	0.50 (0.31, 0.70)	0.52 (0.34, 0.80)	<0.001
Highest temperature	36.2 (36.0, 36.6)	36.2 (36.0, 36.6)	36.2 (36.0, 36.7)	0.7
**Complications at admission**				
Vasopressors	364 (3.3%)	0 (0%)	364 (33%)	
Noninvasive ventilation	311 (2.8%)	0 (0%)	311 (28%)	
Mechanical ventilation	86 (0.8%)	0 (0%)	86 (7.7%)	
Renal replacement therapy	60 (0.5%)	0 (0%)	60 (5.4%)	
**Complications during ICU stay**				
Noninvasive ventilation	437 (4.0%)	0 (0%)	437 (39%)	
Vasopressors	430 (3.9%)	0 (0%)	430 (38%)	
Transfusion	353 (3.2%)	0 (0%)	353 (32%)	
Mechanical ventilation	166 (1.5%)	0 (0%)	166 (15%)	
Renal replacement therapy	103 (0.9%)	0 (0%)	103 (9.2%)	
High-flow nasal cannula	23 (0.2%)	0 (0%)	23 (2.1%)	
**Outcomes **				
ICU LOS, days	3 (2, 4)	3 (2, 4)	4 (3, 7)	<0.001
Hospital LOS, days	5 (3, 7)	5 (3, 6)	7 (5, 11)	<0.001
60-day ICU mortality	109 (1.0%)	12 (0.1%)	97 (8.7%)	<0.001
60-day hospital mortality	157 (1.4%)	37 (0.4%)	120 (11%)	<0.001

**Table 2:** Univariable regression and Multivariable logistic regression to analyze the influence of risk factors of dengue complication.

Feature	Univariable regression			Multivariable regression	
	OR (95% CI)	*P*-value		OR (95% CI)	*P*-value
**Sex (ref. M)**		0.500			
M					
F	0.96 (0.85, 1.08)	0.500			
**Age**		<**0.001**			<**0.001**
16-19 (ref.)					
20-39	1.55 (1.06, 2.36)	0.031		1.43 (0.97, 2.19)	0.082
40-49	1.53 (1.03, 2.36)	0.045		1.31 (0.87, 2.03)	0.200
50-59	1.94 (1.29, 3.00)	0.002		1.56 (1.03, 2.43)	0.044
60-69	2.85 (1.91, 4.39)	<0.001		2.07 (1.36, 3.24)	0.001
70-79	3.45 (2.32, 5.31)	<0.001		2.17 (1.42, 3.43)	<0.001
80+	5.59 (3.77, 8.61)	<0.001		3.10 (2.02, 4.92)	<0.001
**Arterial hypertension**	1.96 (1.72, 2.22)	<**0.001**		1.07 (0.91, 1.27)	0.400
**Diabetes**	1.97 (1.70, 2.29)	<**0.001**		1.19 (1.00, 1.42)	**0.047**
**Cardiovascular disease**	2.28 (1.90, 2.71)	<**0.001**		1.43 (1.14, 1.78)	**0.002**
**Malignancy**	2.67 (2.11, 3.34)	<**0.001**		1.66 (1.26, 2.18)	<**0.001**
**Cerebrovascular disease**	3.20 (2.51, 4.06)	<**0.001**		1.91 (1.46, 2.49)	<**0.001**
**Asthma**	1.15 (0.79, 1.63)	0.400			
**Chronic kidney disease**	5.12 (3.96, 6.58)	<**0.001**		2.94 (2.22, 3.89)	<**0.001**
**Congestive heart failure**	2.30 (1.59, 3.23)	<**0.001**		0.72 (0.47, 1.10)	0.140
**Immunosuppression**	2.76 (2.03, 3.69)	<**0.001**		1.44 (1.00, 2.05)	**0.043**
**Obesity**	2.32 (1.67, 3.15)	<**0.001**		2.22 (1.55, 3.08)	<**0.001**
**Tobacco consumption**	2.00 (1.40, 2.79)	<**0.001**		1.55 (1.06, 2.21)	**0.020**
**Liver cirrhosis**	4.49 (2.35, 8.24)	<**0.001**		3.65 (1.82, 7.04)	<**0.001**
**Platelets class**		<**0.001**			<**0.001**
50-100 cells/mm^3^ (ref.)					
>150,000 cells/mm^3^	1.76 (1.47, 2.10)	<0.001		1.46 (1.21, 1.76)	<0.001
100,000-150,000 cells/mm^3^	1.33 (1.10, 1.60)	0.003		1.46 (1.21, 1.76)	<0.001
<50,000 cells/mm^3^	2.12 (1.79, 2.51)	<0.001		2.25 (1.89, 2.68)	<0.001
**Leukocyte class**		<**0.001**			<**0.001**
<2500 cells/mm^3^ (ref.)					
2500-5000 cells/mm^3^	1.41 (1.16, 1.71)	<0.001		1.10 (0.90, 1.35)	0.400
5000-7000 cells/mm^3^	2.05 (1.63, 2.58)	<0.001		1.62 (1.28, 2.05)	<0.001
>7000 cells/mm^3^	3.43 (2.82, 4.18)	<0.001		2.47 (2.02, 3.03)	<0.001

## References

[1] Organization WH (2009). Dengue: guidelines for diagnosis, treatment, prevention and control.

[2] Cattarino L, Rodriguez-Barraquer I, Imai N, Cummings DAT, Ferguson NM (2020). Mapping global variation in dengue transmission intensity. Sci Transl Med.

[3] World Health Organization (2024). Global dengue surveillance.

[4] Paraná VC, Feitosa CA, da Silva GCS, Gois LL, Santos LA (2024). Risk factors associated with severe dengue in Latin America: a systematic review and meta-analysis. Trop Med Int Health.

[5] Copaja-Corzo C, Flores-Cohaila J, Tapia-Sequeiros G, Vilchez-Cornejo J, Hueda-Zavaleta M, Vilcarromero S (2024). Risk factors associated with dengue complications and death: a cohort study in Peru. PLOS ONE.

[6] Carras M, Maillard O, Cousty J, Gérardin P, Boukerrou M, Raffray L (2023). Associated risk factors of severe dengue in Reunion Island: a prospective cohort study. PLoS Negl Trop Dis.

[7] von Elm E, Altman DG, Egger M, Pocock SJ, Gøtzsche PC, Vandenbroucke JP (2007). The Strengthening the Reporting of Observational Studies in Epidemiology (STROBE) statement: guidelines for reporting observational studies. The Lancet.

[8] Soares M, Borges LP, Bastos L dos SL, Zampieri FG, Miranda GA, Kurtz P (2024). Update on the Epimed Monitor Adult ICU Database: 15 years of its use in national registries, quality improvement initiatives and clinical research. Crit Care Sci.

[9] Peres IT, Hamacher S, Cyrino Oliveira FL, Bozza FA, Salluh JIF (2022). Data-driven methodology to predict the ICU length of stay: a multicentre study of 99,492 admissions in 109 Brazilian units. Anaesth Crit Care Pain Med.

[10] Peres IT, Hamacher S, Oliveira FLC, Bozza FA, Salluh JIF (2021). Prediction of intensive care units length of stay: a concise review. Rev Bras Ter Intensiva.

[11] Kim J-H (2009). Estimating classification error rate: repeated cross-validation, repeated hold-out and bootstrap. Comput Stat Data Anal.

[12] Kuhn M, Johnson K, Kuhn M, Johnson K (2013). Applied predictive modeling.

[13] Walsh CG, Sharman K, Hripcsak G (2017). Beyond discrimination: a comparison of calibration methods and clinical usefulness of predictive models of readmission risk. J Biomed Inform.

[14] White IR, Royston P, Wood AM (2011). Multiple imputation using chained equations: issues and guidance for practice. Stat Med.

[15] Garcia-Gallo E, Edinburgh T, Bastos L, Peres I, Imtiaz H, Raffaini LE, Duque-Vallejo S, Pesonel E, Merson L (2024). ISARIC VERTEX (v1.0). ISARIC.

[16] Thach TQ, Eisa HG, Hmeda AB, Faraj H, Thuan TM, Abdelrahman MM (2021). Predictive markers for the early prognosis of dengue severity: a systematic review and meta-analysis. PLoS Negl Trop Dis.

[17] Moallemi S, Lloyd AR, Rodrigo C (2023). Early biomarkers for prediction of severe manifestations of dengue fever: a systematic review and a meta-analysis. Sci Rep.

[18] Chagas GCL, Rangel AR, Noronha LM, Veloso FCS, Kassar SB, Oliveira MJC (2022). Risk factors for mortality in patients with dengue: a systematic review and meta-analysis. Trop Med Int Health.

[19] Díaz-Quijano FA (2008). Predictors of spontaneous bleeding in dengue patients: a systematic review of the literature. Invest Clín.

[20] Kallas EG, D’Elia Zanella LGFAB, Moreira CHV, Buccheri R, Diniz GBF, Castiñeiras ACP (2019). Predictors of mortality in patients with yellow fever: an observational cohort study. Lancet Infect Dis.

[21] Shepard DS, Undurraga EA, Halasa YA, Stanaway JD (2016). The global economic burden of dengue: a systematic analysis. Lancet Infect Dis.

[22] Limkittikul K, Brett J, L’Azou M (2014). Epidemiological trends of Dengue disease in Thailand (2000–2011): a systematic literature review. PLoS Negl Trop Dis.

[23] Caicedo-Torres W, Paternina Á, Pinzón H, Montes y Gómez M, Escalante HJ, Segura A, de D Murillo J (2016). Advances in artificial intelligence—IBERAMIA 2016.

[24] Huang S-W, Tsai H-P, Hung S-J, Ko W-C, Wang J-R (2020). Assessing the risk of dengue severity using demographic information and laboratory test results with machine learning. PLoS Negl Trop Dis.

[25] Madewell ZJ, Rodriguez DM, Thayer MB, Rivera-Amill V, Paz-Bailey G, Adams LE (2025). Machine learning for predicting severe dengue in Puerto Rico. Infect Dis Poverty.

[26] Sarma D, Hossain S, Mittra T, Bhuiya MdAM, Saha I, Chakma R (2020). Dengue prediction using machine learning algorithms.

