# Supplementary material for: Clinical characteristics, complications and outcomes of critically ill patients with Dengue in Brazil, 2012-2024: A nationwide, multicenter cohort study
Source: Int J Infect Dis. Author manuscript; Available in PMC 2026 Feb 4. (PMC7618716; doi:10.1016/j.ijid.2025.108023)
Supplement: Supplementary Information [file EMS212280-supplement-Supplementary_Information.pdf]

Supplementary materials

Supplementary material associated with this article can be found, in the online version, at doi:10.1016/j.ijid.2025.108023.
